# The role of multifocality in predicting central lymph node metastasis in initially treated 18-55 years old female patients with unilateral papillary thyroid microcarcinoma

**DOI:** 10.3389/fonc.2023.1197232

**Published:** 2023-08-31

**Authors:** Li Zhao, Han Li, Yuqin He, Yixuan Song, Ziren Kong, Yang Liu, Jian Wang, Yiming Zhu, Dangui Yan, Shaoyan Liu

**Affiliations:** National Cancer Center/National Clinical Research Center for Cancer/Cancer Hospital, Chinese Academy of Medical Sciences and Peking Union Medical College, Department of Head and Neck Surgery, Beijing, China

**Keywords:** papillary thyroid carcinoma, papillary thyroid micro carcinoma, cervical lymph node metastasis, multifocality, female Beijing natural science foundation (grant no.7232351), special

## Abstract

**Objective:**

To determine the predictive ability of multifocality for central lymph node metastasis in initially treated 18-55 years old female patients with unilateral papillary thyroid microcarcinoma.

**Study design:**

Retrospective review.

**Setting:**

Tertiary medical center.

**Methods:**

We retrospectively collected clinical data from initially treated papillary thyroid microcarcinoma (PTMC) patients at Cancer Hospital Chinese Academy of Medical and sciences between January 1st, 2018, and December 31st, 2018. Data from 975 initially treated 18-55 years old female patients with unilateral PTMC was collected. We also collected data from 340 initially treated 18-55 years old male patients with unilateral PTMC patients to compare the results between genders. Clinicopathological factors associated with central lymph node metastasis (CLNM) were investigated by univariate and multivariate analysis.

**Results:**

(1) In the female group, there were 196 (20.1%) cases that had tumor multifocality, including 126 (12.9%) with 2 foci and 70 (7.2%) with >2 foci. The risk of CLNM in patients with 2 foci was not significantly higher than patients with 1 focus (37.3% vs 38.6%, P=0.775). However, diagnosed with >2 foci were independently and positively correlated with CLNM (OR=2.708, 95%CI=1.592-4.607, P<0.001), as was tumor diameter >0.55cm (OR=2.047, 95%CI=1.535-2.730, P<0.001). (2) In the male group, the risk of CLNM with 2 foci was significantly higher than 1 focus (P=0.008). Compared to female patients, the risk of CLNM was significantly higher in patients with 1 focus (P<0.001) or 2 foci (P<0.001).

**Conclusion:**

In summary, the risk of CLNM in patients with 2 foci was not significantly higher than patients with 1 focus, while multifocality with over 2 foci was an independent risk factor of CLNM. Therefore, multifocality in this subgroup should not be simply defined as “more than 1 focus”. Future models that include multifocality as a predictive factor for cervical lymph node metastasis could consider stratifying the cohort into smaller subgroups for more accurate conclusions.

## 1 Introduction

Papillary thyroid carcinoma (PTC) is an epithelial malignancy originating from the follicular cell of the thyroid gland. The incidence of PTC increased dramatically in the last decades. Overdiagnosis might have contributed to this process (1, 2), which, in turn, can lead to overtreatment (3). Radical surgery is the most important therapy for PTC. Besides surgery, active surveillance was introduced in the 2015 American Thyroid Association guidelines as an alternative method to manage very low-risk tumors (4). However, active surveillance requires the identification of patients with progression risk. Multifocality has been considered as a potential risk factor for poorer prognosis in PTC which has been the subject of several papers in recent years. Some suggest that multifocality has no independent risk prognostic value (5), while some consider multifocality as a significant risk factor for disease progression and recurrence (6). Additionally, a meta-analysis by Mao et al. found that multifocality, along with age, gender, tumor size and extra-thyroidal extension (ETE), was significantly associated with cervical lymph node metastasis (7). Moreover, it has been observed that cervical lymph node metastases are associated with compromised survival in young patients (8) and young female patients, compared to elder or male patients, are more seen for PTC (9). To avoid bias originated from age and tumor size, we started a cohort only including initially treated 18-55 years old patients with unilateral papillary thyroid microcarcinoma (PTMC), defined as a PTC measuring ≤ 1.0 cm in greatest dimension, to further explore the relevance between multifocality and cervical lymph node metastases in this population.

## 2 Materials and methods

### 2.1 Patients and study design

A retrospective study was conducted on a primary cohort of patients that were pathologically diagnosed with PTC at the Cancer Hospital Chinese Academy of Medical Sciences (Beijing, China) between January 1^st^, 2018, and December 31^st^, 2018. The inclusion criteria were as follow: 1) Initially treated for PTMC. 2) Age: 18-55 years old. 3) Postoperative pathology report was available. 4) Unilateral PTC. The exclusion criteria were as follow: 1) Patients with highly advanced locoregional focus(T4b). 2) Patients with distant metastasis(M1). 3) Patients with high−risk histology (eg, poorly differentiated, tall cell, columnar cell, hobnail variants, diffuse sclerosing, and insular). The postoperative pathology reports were reviewed by two independent pathologists. The results were recorded according to the official report issued, we obtained information on tumor size, multifocality, histology, extra-thyroidal extension (ETE), cervical nodal metastases, Hashimoto’s disease (HD), perineural invasion (PNI) and lymphovascular invasion (LVI). Diagnose of distance metastasis was based on the results of all available preoperative examinations including clinical examination, blood tests, ultrasound of the neck and abdomen, CT of neck and chest, and bone scintigraphy. Disease staging was classified according to the 8th edition of the American Joint Cancer Committee (AJCC) system.

The maximum Youden’s Index was calculated for cutpoint analysis to determine optimal cutpoint for tumor size. Youden’s Index (J) is calculated as: J = Sensitivity + Specificity −1, with a value of 1 representing a perfect test with no false positives or false negatives. The maximum Youden’s Index is the point on the ROC curve where resultant J value is closest to 1.

### 2.2 Surgery strategy

In our institution, surgical managements have been performed according to the 2015 American Thyroid Association Management Guidelines. Lobectomy plus ipsilateral central lymph node dissection (CLND) was typically performed as initial surgical treatment for PTMC patients with malignant foci that were limited to a single lobe. When malignant foci had extra thyroidal extension (ETE) or multifocal tumors limited to a single lobe, a total thyroidectomy plus ipsilateral CLND was performed. Lateral lymph node dissection (LLND), including level II-V, was performed only in cases with clinical evident lateral neck lymph node metastasis (LLNM).

### 2.3 Statistical analysis

Statistical analyses to identify risk factors were performed using SPSS (version 24.0; IBM Corp., Armonk, NY, USA). Categorical variables were grouped based on clinical findings, and decisions on the groups were made before modeling. Descriptive statistics were used to report categorical data. Continuous data are reported as median and interquartile range. Group differences were analyzed using the χ2 test or Fisher’s exact test. Continuous variables were compared using the t-test, Mann–Whitney U test and Kruskal-Wallis H test for variables with skewed distribution. Logistic regression analysis was performed for multivariate analyses.

This retrospective study was reviewed and approved by the institutional review board of the National Cancer Hospital of the Chinese Academy of Medical Sciences.

## 3 Results

### 3.1 Characteristics of female patients

**Figure 1:**
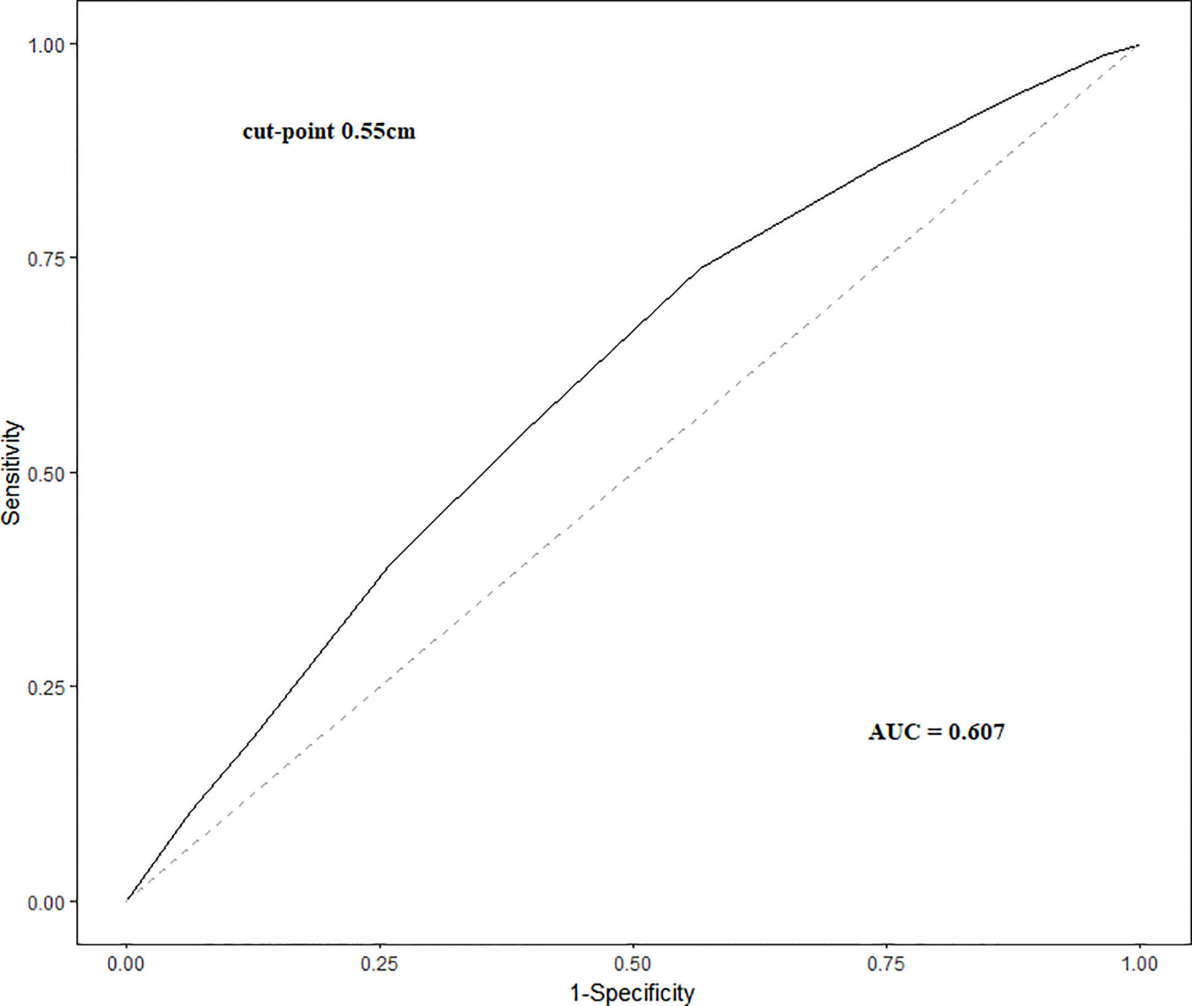
The optimal cutpoint for tumor size in predicting central lymph node metastasis.

A total of 975 female patients were identified according to our inclusion criteria. One patient was further excluded because of high−risk histology. The characteristics of the whole cohort are shown in **Table 1**. In the whole cohort, the median age was 42 (35-49), 620 (63.6%) patients received lobectomy while 355 (36.4%) received total thyroidectomy. Median pathologically measured Maximum tumor diameter of the primary focus was 0.6 (0.5-0.8) cm. 196 (20.1%) patients were multifocal, including 126 (12.9%) with 2 foci and 70 (7.2%) with more than 2 foci. Microscopic ETE (mETE) was diagnosed in 358 (36.7%) patients and a total of 395 (40.5%) patients were diagnosed with CLNM. The primary tumor size was larger in patients with CLNM, with an area under the ROC curve (AUC) of 0.607 (95%CI=0.571-0.643, P<0.001) (**Figure 1**). The maximum Youden’s index indicated tumor size of 0.55cm as the optimal cutpoint.

**Table 1:** Patient clinical characteristics.

Characteristic	N (%) ^a^
Age, in median years (IQR)	42 (35-49)
Surgery	
Lobectomy	620 (63.6%)
Total thyroidectomy	355 (36.4%)
Pathology	
Maximum diameter of primary focus,	
in median cm (IQR)	0.6 (0.5-0.8)
≤0.55cm	356 (36.5%)
>0.55cm	619 (63.5%)
Number of foci	
1	779 (79.9%)
2	126 (12.9%)
>2	70 (7.2%)
mETE	358 (36.7%)
central lymph node metastasis	395 (40.5%)
HD	268 (27.5%)
Perineural invasion	14 (1.0%)
Lymphovascular invasion	10 (1.4%)

a unless otherwise specified. IQR, interquartile range; mETE, microscopic extra-thyroidal extension; HD, hashimoto's disease.

### 3.2 Results of patients with 1 focus and 2 foci in the female group

First, we compared the clinical features of patients with 1 focus or 2 foci, a total amount of 905 were identified. We compared the clinical features between patients with or without CLNM, results are shown in **Table 2**. Maximum tumor diameter >0.55cm was a risk factor for CLNM (OR=2.047, 95%CI=1.535-2.730, P<0.001) and showed statistical difference in both univariate and multivariate analysis, while mETE only showed significant difference in univariate analysis. Besides, HD, PNI and LVI were not identified as risk factors for CLNM in univariate analysis. Interestingly, the risk of CLNM in patient with 1 focus was 38.6% (301/779) which was not significantly different from patients with 2 foci 37.3% (47/126) (P=0.775). Therefore, we combined the patients with 1 focus and 2 foci in further data analysis.

**Table 2:** Clinical features and their correlation with central lymph node metastasis in patients with 1 lesion or 2 lesions.

Clinical Feature	N (%)^a^	Univariate Analysis			Multivariate Analysis		
		OR	95% CI	P value	OR	95% CI	P value
Pathology							
Maximum diameter of the primary tumor (>0.55cm vs ≤0.55cm)	251 (44.7%) / 97 (28.3%)	2.047	1.535 - 2.730	<0.001	1.961	1.465 - 2.626	<0.001
mETE (with vs without)	144 (44.0%) / 204 (35.3%)	1.443	1.094 - 1.903	0.009	1.293	0.974 - 1.717	0.076
Number of foci (>2 vs ≤2)	47 (37.3%) / 301 (38.6%)	0.945	0.640 - 1.394	0.775	--	--	--
HD (with vs without)	90 (25.9%) / 157 (28.2%)	0.889	0.657 - 1.203	0.445	--	--	--
PNI (with vs without)	5 (41.7%) / 343 (38.4%)	1.145	0.361 - 3.637	0.818	--	--	--
LVI (with vs without)	4 (80.0%) / 344 (38.2%)	6.465	0.720 - 58.082	0.055	--	--	--

mETE, microscopic extra-thyroidal extension; HD, hashimoto's disease; PNI, perineural invasion; LVI, lymphovascular invasion.

a Percentages are based on the total number of patients with each characteristic in the study group.

### 3.3 Results after combining patients with 1 focus and 2 foci in the female group

The results of clinical features and their correlation with CLNM in the whole cohort are shown in **Table 3**. Maximum tumor diameter >0.55cm was still a significant risk factor for CLNM (OR=1.951, 95%CI=1.468-2.592, P<0.001), as was multifocality with over 2 foci (OR=2.708, 95%CI=1.592-4.607, P<0.001). LVI and mETE was identified as risk factors only in univariate analysis. HD and PNI did not show statistical significance in both univariate and multivariate analysis.

**Table 3:** Clinical features and their correlation with central lymph node metastasis.

Clinical Feature	N (%)^a^	Univariate Analysis			Multivariate Analysis		
		OR	95% CI	P value	OR	95% CI	P value
Pathology							
Maximum diameter of the primary tumor (>0.55cm vs ≤0.55cm)	291 (47.0%) / 104 (29.2%)	2.150	1.629 - 2.838	<0.001	1.951	1.468 - 2.592	<0.001
mETE (with vs without)	167 (46.6%) / 228 (37.0%)	1.492	1.145 - 1.943	0.003	1.299	0.987 - 1.710	0.062
Number of foci (>2 vs ≤2)	47 (67.1%) / 348 (38.5%)	3.271	1.951 - 5.482	<0.001	2.708	1.592 - 4.607	<0.001
HD (with vs without)	104 (38.8%) / 291 (41.2%)	1.103	0.827 - 1.471	0.504	--	--	--
PNI (with vs without)	7 (50.0%) / 388 (40.4%)	1.477	0.514 - 4.244	0.466	--	--	--
LVI (with vs without)	9 (90.0%) / 386 (40.0%)	13.500	1.704 - 106.984	0.001	7.315	0.897 - 59.659	0.063

mETE, microscopic extra-thyroidal extension; HD, hashimoto's disease; PNI, perineural invasion; LVI, lymphovascular invasion.

a Percentages are based on the total number of patients with each characteristic in the study group.

### 3.4 Comparison with male patients in the same time frame

We further identified 340 initially treated 18-55 years old male patients with unilateral papillary thyroid microcarcinoma in the same time frame to compare our results between genders. In this group, 266 (78.2%) patients were with only 1 focus, 42 (12.4%) with 2 foci and 32 (9.4%) with more than 2 foci, CLNM rate was 54.5% (145/266), 76.2% (32/42) and 71.9% (23/32) respectively. The risk of CLNM in patients with 2 foci was significantly higher than patients with 1 focus (P=0.008). The distribution of numbers of foci was similar in both sexes (**Table 4**). Compared to female patients, the CLNM rate was significantly higher in patients with 1 focus and 2 foci while CLNM rate was not significantly different in patients with more than 2 foci (**Table 5**).

**Table 4:** Comparison of CLNM rate between male and female.

Multifocality	Gender		P value
	Male	Female	
1 focus	54.5% (145/266)	38.6% (301/779)	<0.001
2 foci	76.2% (32/42)	37.3% (47/126)	<0.001
>2 foci	71.9% (23/32)	67.1% (47/70)	0.633

**Table 5:** Comparison of lesion number distribution between male and female.

Multifocality	Gender		P value
	Male	Female	
1 focus	266 (78.2%)	779 (79.9%)	0.412
2 foci	42 (12.4%)	126 (12.9%)	--
>2 foci	32 (9.4%)	70 (7.2%)	--

## 4 Discussion

The incidence of PTMC has risen rapidly in the last decade, due to the large amount of PTC patients receiving initial surgery treatment in our hospital, we were able to evaluate the role of multifocality in a very specific subgroup and a short time frame but still in a relatively large population, which can relatively reduce study bias. This study included 975 ipsilateral PTMC patients which might further reveal the role of multifocality in 18-55 years old female patients and 340 male patients to compare the results between genders.

The rate of CLNM in our population was 40.5% (395/975) while other published articles reported a rate of CLNM in 24% to 64% (10–12). Although most patients with PTMC have a good prognosis, PTMC patients with CLNM may suffer from recurrence and poor prognosis (10, 13).

Multifocality, mostly defined as >1 focus, has been identified as an independent risk factor for CLNM in PTMC in several previous studies (14–18). However, different number of foci may perform different patterns of lymphatic metastasis. In our study, female patients with >2 foci had a higher risk of CLNM compared to female patients with 2 tumor foci. This result is in line with the study by Feng et al. (19) and a previous meta-analysis (20), showing that multifocality is a risk factor for CLNM. However, 2 foci were not a risk factor in our study, which was different from these two studies.

An important factor in diagnosing multifocality is to identify whether the multiple foci have different clonal origins (true multicentricity). It is also possible that multifocality is the result of metastasis and spread of a single focus in the thyroid gland. Intra-thyroidal metastases from a single focus indicates progress of disease while true multicentricity does not necessarily represent progress of disease as a function of time. Therefore, the reason why female patients with 1 focus and 2 foci have the similar CLNM rate might because that most 18-55 years old female PTMC patients with 2 foci are true multicentric or don’t have enough time to develop CLNM. However, the development of more than 2 foci is more likely the result of intra-thyroidal metastases from a single focus, representing longer time of disease progression.

Another possible reason for the result in our study is that we only included PTMC patients, which means that the tumor size of the secondary focus is ≤ 1.0cm. Larger size of the secondary focus could be another risk factor for CLNM. More data and further studies are needed to validate this hypothesis.

Furthermore, the meaning of multifocality is different between genders in our study. Male patients with 2 foci have higher rates of CLNM compared to those with a single focus. While the distribution of numbers of focus in male patients were similar compared to female patients, male patients with 1 focus and 2 foci have higher CLNM rates. Indicating that male patients have more aggressive disease in these subgroups. However, this difference could not be seen in patients with more than 2 foci.

Whether the multiple foci are unilateral or bilateral should also be considered. Our study only included patients with unilateral foci which could reduce bias originated from this characteristic. A previous study has already shown that PTC patients with unilateral multifocality had a higher risk of CLNM compared to those with bilateral multifocality (21).

There are several potential limitations in our study. First, this is retrospective research, which is inevitable not randomized. Second, the diagnosis of bilateral or unilateral PTC is partially influenced by the surgery (lobectomy or total thyroidectomy) the patient received, there are potential bilateral PTC patients in this cohort which could not have been diagnosed before initial surgery. Third, the diagnoses of multifocality relied on postoperative pathological reports. Although our result demonstrated enough predictive ability in multivariate regression, its application in clinical practice, especially in preoperative evaluation of surgical approach, remains difficult. Finally, our data only analyzed the role of multifocality in a specific subgroup, whether other patients, such as patients with larger tumors, have the same characteristics needs to be validated in future research.

## 5 Conclusion

In our study, the risk of CLNM in female patients with 2 foci was not significantly higher than those with 1 focus, while multifocality with over 2 foci was an independent risk factor of CLNM. Therefore, multifocality in this subgroup should not be simply defined as “more than 1 focus”. Future models that include multifocality as a predictive factor for cervical lymph node metastasis could consider stratifying the cohort into smaller subgroups for more accurate conclusions.

## Data availability statement

The raw data supporting the conclusions of this article will be made available by the authors, without undue reservation.

## Ethics statement

The studies involving human participants were reviewed and approved by institutional ethics committee of the Cancer Hospital, Chinese Academy of Medical Sciences. The patients/participants provided their written informed consent to participate in this study. Written informed consent was obtained from the individual(s) for the publication of any potentially identifiable images or data included in this article.

## Author contributions

LZ: Study concepts, study design, data analysis, manuscript preparation. HL: data interpretation and manuscript preparation. YH: data acquisition and interpretation. YS and ZK: data acquisition. YL: statistical analysis. JW: data acquisition, data analysis and interpretation. YZ: quality control of data and algorithms. DY: quality control of data and algorithms. SL: Study concepts, study design, manuscript review.
